# Epidemiological analysis of sexually transmitted infections pathogens and related microorganisms in South Korea: coinfection patterns and pathogen correlations

**DOI:** 10.1186/s12879-026-13232-7

**Published:** 2026-04-07

**Authors:** Jeonghyun Kim, Jinhee Cho, Unyeong Go, Gwi-young Oh

**Affiliations:** 1 Laboratory Medicine, EONE Laboratories, Incheon, South Korea; 2 Infectious Disease Research Center, Green Cross Laboratories, Yongin-si, South Korea

**Keywords:** Sexually transmitted infections, Coinfection, Lift index, Phi coefficient, Association rule mining, Heatmaps

## Abstract

**Background:**

Sexually transmitted infections (STIs) affect hundreds of millions of people worldwide each year and remain a major public health concern. The adoption of multiplex PCR, which allows simultaneous detection of multiple pathogens, has led to the accumulation of large-scale data on STI coinfections. In this study, we analyzed multiplex PCR results for 12 microorganisms included in a commercial STI testing panel to characterize epidemiological patterns in South Korea and to quantitatively evaluate pathogen–pathogen coinfection relationships using lift index and phi coefficient.

**Methods:**

We retrospectively analyzed multiplex PCR results for 12 microorganisms included in an STI testing panel — Neisseria gonorrhoeae (*N. gonorrhoeae*), Chlamydia trachomatis (*C. trachomatis*), Mycoplasma hominis (*M. hominis*), Trichomonas vaginalis (*T. vaginalis*), Ureaplasma urealyticum (*U. urealyticum*), Mycoplasma genitalium (*M. genitalium*), Herpes simplex virus type 1 (HSV-1) and type 2 (HSV-2), Gardnerella vaginalis (*G. vaginalis*), Treponema pallidum (*T. pallidum*), Candida albicans (*C. albicans*), and Ureaplasma parvum (*U. parvum*)— from individuals aged 18–90 years tested at Eone Laboratories between January 2023 and December 2024. DNA was extracted using the Nextractor^®^ NX-48 system, and PCR was performed with the ElsiQ STI-12 Detection Kit. Pathogen positivity rates were assessed by sex, age, and specimen type. Association rule mining was applied to evaluate coinfection patterns using the lift index and phi coefficient, and the results were visualized as heatmaps.

**Results:**

A total of 1,105,261 samples were analyzed, with an overall positivity rate of 8.67% among all individual pathogen-specific tests. At the specimen level, 43.6% were negative, 26.1% were positive for a single pathogen, and 30.4% had coinfections. Compared with males, females presented a greater rate of coinfections. For most pathogens, positivity rates were highest in individuals under the age of 30 years and tended to decrease with age. For some pathogens, a bimodal pattern of positivity was observed in females, with a second increase occurring around the age of 50. Among female samples, vaginal swabs presented higher positivity rates than did urine samples for all pathogens. Analysis of coinfection patterns revealed that the lift index tended to be greater for pathogen pairs with low individual positivity rates, whereas the phi coefficient was relatively higher for pathogens with higher positivity rates. Pathogen pairs that presented a lift index ≥ 3 in both male and female samples included *N. gonorrhoeae–C. trachomatis*,* M. hominis–T. vaginalis*,* T. pallidum–T. vaginalis*, and *T. pallidum–C. trachomatis*, whereas *U. parvum–M. hominis* and *U. urealyticum–M. hominis* presented phi coefficients ≥ 0.1 in both sexes.

**Conclusions:**

This study provides quantitative insights into the epidemiological characteristics and inter-pathogen coexistence of STI-related microorganisms in South Korea using multiplex PCR–derived data and association metrics, including the lift index and phi coefficient. These findings may serve as foundational data for refining future STI surveillance strategies and for further investigations into the mechanisms underlying coinfections.

**Clinical trial number:**

Not applicable.

## Introduction

Sexually transmitted infections (STIs), including asymptomatic cases, are diseases transmitted through sexual contact and affect hundreds of millions of people worldwide each year, posing a significant public health burden. The World Health Organization (WHO) periodically reports global estimates of the prevalence and incidence of major treatable STIs, including syphilis, chlamydia, gonorrhea, and trichomoniasis, with estimates released approximately every four years. Notably, WHO data updated in 2022 estimated that approximately 8 million adults aged 15–49 years were infected with syphilis worldwide [1].

Moreover, in the Republic of Korea, the following seven STIs are designated notifiable infectious diseases: syphilis, gonorrhea, chlamydia, chancroid, genital herpes, condyloma acuminata, and human papillomavirus infection. Since 2024, the surveillance system for syphilis has changed from sentinel surveillance to mandatory case-based reporting, while the remaining STIs are managed through a sentinel surveillance system [2]. According to recent national surveillance reports from South Korea, the number of HPV cases has increased by approximately 30% through 2024, and the prevalence of primary syphilis has sharply increased to approximately 46% by 2023. These trends indicate that STIs remain a persistent public health challenge [3].

Diagnostic methods for STIs include nucleic acid amplification tests (NAATs), culture and serological tests. Among these methods, culture and serological methods are limited by long turnaround times or low sensitivity. Since many STI cases are asymptomatic and often involve coinfections, multiplex PCR has been increasingly adopted in recent years [4]. Several commercially available multiplex PCR kits are currently used in clinical practice, including the Cepheid GeneXpert^®^ STI Panel, Roche cobas^®^ STI Assay, Abbott RealTime STI Panel, Seegene Anyplex™ II STI-12, Kogene Biotech PowerChek™ STI Multiplex Real-time PCR Kit, and the SolGent Solg™ 2X Multiplex PCR Series. In South Korea, National Health Insurance reimbursement for diagnostic testing is determined by whether a test panel includes STI pathogens designated in the reimbursement guidelines. Under this reimbursement framework, a variety of multiplex PCR assays that encompass a broader range of pathogens within the same reimbursement category are widely used in routine clinical practice.

As the use of multiplex PCR in STI diagnostics has expanded, interest in pathogen coinfection patterns has also increased. However, most previous studies have primarily reported overall coinfection rates, with limited investigation into specific pathogen–pathogen associations [5, 6].

To better elucidate these interpathogen relationships, association rule mining (ARM) can be employed. ARM is a data-mining technique originally developed for market basket analysis to identify associations among items that frequently co-occur. This approach is useful for quantitatively evaluating relationships between binary variables, using indices such as support, which represents the frequency of co-occurrence; confidence, which indicates the conditional probability of co-occurrence; and lift, which reflects the strength of association relative to chance. Among these, lift is particularly helpful for comparing pathogen–pathogen relationships because it reflects how much the observed co-occurrence exceeds what would be expected under statistical independence. However, lift may overestimate associations when both items are rare, and the ϕ (phi) coefficient, which is derived from Pearson’s correlation, has been proposed as a complementary measure. These association metrics have increasingly been applied in healthcare research to analyze large-scale clinical datasets, including studies of disease co-occurrence, disease–symptom relationships, and disease–drug interactions [7–9].

This study aimed to characterize epidemiological patterns of 12 STI-related microorganisms in South Korea by assessing positivity rates according to sex, age, and specimen type using a large-scale dataset of multiplex PCR results, and quantitatively evaluate pathogen–pathogen coinfection relationships by applying association rule mining metrics, including the lift index and phi coefficient.

## Materials and methods

### Study design

This study retrospectively analyzed multiplex PCR results for 12 sexually transmitted infection (STI)-related microorganisms, Neisseria gonorrhoeae (*N. gonorrhoeae*), Chlamydia trachomatis (*C. trachomatis*), Mycoplasma hominis (*M. hominis*), Trichomonas vaginalis (*T. vaginalis*), Ureaplasma urealyticum (*U. urealyticum*), Mycoplasma genitalium (*M. genitalium*), Herpes simplex virus types 1 and 2 (HSV-1, HSV-2), Gardnerella vaginalis (*G. vaginalis*), Treponema pallidum (*T. pallidum*), Candida albicans (*C. albicans*), and Ureaplasma parvum (*U. parvum*), conducted at Eone Laboratories from January 2023 to December 2024, involving individuals aged 18 to 90 years. The dataset included information on patient sex, age, specimen type, and date of receipt. Prior to analysis, records with invalid or missing patient identifiers, invalid age values, or invalid test results were excluded.

Eone Laboratories is a large commercial clinical laboratory in South Korea that receives specimens collected during routine clinical care from diverse healthcare institutions nationwide, ranging from primary to tertiary care settings. Analyses were performed at the specimen level because individual-level identifiers were not available; multiple specimens from the same individual could not be distinguished, and duplicate testing may not be excluded. Specimens classified as “other” consisted mainly of semen samples from male patients, with small numbers of tissue specimens and including those with missing or insufficient specimen information.

### DNA extraction and PCR reaction

Nucleic acids were extracted using the Nextractor^®^ NX-48 system (Genolution, Republic of Korea) in accordance with the manufacturer’s instructions, utilizing either the NX-48 Bacterial DNA Kit or the Urine/Swab DNA Kit (Genolution, Republic of Korea). PCR amplification was performed using the ElsiQ STI-12 Detection Kit 1/2 and 3/4 (Eone Biotech, Republic of Korea). The PCR protocol included a 3-minute uracil‒DNA glycosylase (UDG) reaction at 50 °C, followed by initial activation at 95 °C for 15 min, then 45 cycles of denaturation at 95 °C for 20 s and annealing/extension at 60 °C for 40 s. A cycle threshold (Ct) value of ≤ 40 was considered positive.

### Statistical analysis

Statistical analyses were conducted using Python 3.10.9. For inferential statistics, the statsmodels and scipy.stats libraries were utilized. To compare pathogen positivity rates between specimen types in female samples, unpaired odds ratios (ORs) were calculated using 2 × 2 contingency tables comparing vaginal swabs and urine samples. The 95% confidence intervals (CIs) for ORs were estimated using Woolf’s logit method with a continuity correction of 0.5. Fisher’s exact tests were performed, and *p*-values < 0.05 were considered statistically significant.

Co-occurrence relationships among the pathogens were assessed using the lift index—a key metric in association rule mining (ARM)—and the phi coefficient. Lift was calculated as the joint detection probability of two pathogens divided by the product of their individual detection probabilities, and was used to quantitatively assess the strength of association between pathogen pairs;

$${\rm{Lift}}\left({{\rm{A, B}}} \right){\rm{ = P}}\left({{\rm{A }} \cap {\rm{ B}}} \right){\rm{/}}\left({{\rm{P}}\left({\rm{A}} \right)\,{\rm{\cdot}}\,{\rm{P}}\left({\rm{B}} \right)} \right)$$

The phi coefficient (φ), a measure of association for binary variables, was also calculated based on 2 × 2 contingency tables;

$${\rm{\varphi = }}\left({{\rm{ad - bc}}} \right)/\surd \left({\left({{\rm{a + b}}} \right)\left({{\rm{c + d}}} \right)\left({{\rm{a + c}}} \right)\left({{\rm{b + d}}} \right)} \right)$$

where a, b, c, and d represent the cell counts in a 2 × 2 table.

All data processing was conducted using the pandas library, and results were visualized using heatmaps generated with seaborn.

A lift value greater than 1.0 indicates the presence of a positive association between two pathogens, meaning that the presence of one pathogen increases the likelihood of co-occurrence of the other pathogen beyond what would be expected by chance [10, 11]. In this study, the strength of associations was evaluated using lift thresholds of ≥ 3.0 and > 10.0, and a φ coefficient greater than 0.1 was defined as the criterion for a meaningful association, consistent with Cohen’s guidelines for effect size interpretation [12].

## Results

### Demographic summary

A total of 1,105,261 samples were tested for 12 sexually transmitted infection (STI)-related microorganisms, yielding 13,263,132 individual pathogen-specific test results, of which 1,149,919 results (8.67%) were positive. At the specimen level, 34.0% were from males, and 66.0% were from females. Overall, 43.6% of specimens were negative for all pathogens, 26.1% were positive for a single pathogen, and 30.4% exhibited coinfections. The most frequently tested age group was individuals in their 30s for both sexes, followed by those in their 40s and 20s for females and those in their 20s and 40s for males. Regarding the distribution of specimen types, vaginal swabs accounted for 88.6% of the female samples, whereas urine accounted for 96.6% of the male samples (Table 1).

**Table 1:** Demographic characteristics and infection status of the analyzed specimens

Characteristic	Total	(%)	Female(%)		Male(%)	
**Sex**	1,105,261	(100%)	729,112	(66.0%)	376,149	(34.0%)
**Age group** (years)						
18–19	11,688	(1.1%)	8377	(1.1%)	3311	(0.9%)
20–29	235,313	(21.3%)	157,112	(21.5%)	78,201	(20.8%)
30–39	299,100	(27.1%)	196,270	(26.9%)	102,830	(27.3%)
40–49	224,247	(20.3%)	158,705	(21.8%)	65,542	(17.4%)
50–59	171,275	(15.5%)	119,369	(16.4%)	51,906	(13.8%)
60–69	110,305	(10%)	63,090	(8.7%)	47,215	(12.6%)
70+	53,333	(4.8%)	26,189	(3.6%)	27,144	(7.2%)
**Specimen type**						
Urine	443,696	(40.1%)	80,253	(11%)	363,443	(96.6%)
Vaginal swab	646,838	(58.5%)	646,210	(88.6%)	-	
other	14,727	(1.3%)	2649	(0.4%)	12,706	(3.4%)
**Coinfection status** (number of simultaneously detected STI-related microorganisms)						
All-negative cases	481,437	(43.6%)	219,295	(30.1%)	262,142	(69.7%)
Single Infection	288,093	(26.1%)	200,006	(27.4%)	88,087	(23.4%)
Multiple infections	335,731	(30.3%)	309,811	(42.5%)	25,920	(6.8%)
2 infections	198,821	(18%)	177,237	(24.3%)	21,584	(5.7%)
3 infections	94,936	(8.6%)	91,200	(12.5%)	3736	(1%)
4 + infections	41,974	(3.7%)	41,374	(5.7%)	600	(0.1%)

### Pathogen-specific positivity rates

Table 2 presents the number of positive cases and positivity rates for each pathogen by sex. Among females, the five pathogens with the highest positivity rates were *G. vaginalis *(53.2%), *U. parvum *(37.5%), *C. albicans *(16.3%), *U. urealyticum *(14.6%), and *M. hominis *(8.8%).

In males, *U. urealyticum *(12.4%), *U. parvum *(10.3%), *C. trachomatis *(4.7%), *M. hominis *(3.9%), *and M. genitalium *(2.6%) had the highest positivity rates. The remaining pathogens had low positivity rates of 2% or less.

**Table 2:** Sex-specific positivity rates (%) and number of positive cases for 12 sexually transmitted infection (STI)-related microorganisms

Pathogen	Total Positive Cases(%)		Female Positive Cases(%)		Male Positive Cases(%)	
*Treponema pallidum*	210	(0.02%)	70	(0.01%)	140	(0.04%)
*Trichomonas vaginalis*	2591	(0.23%)	2217	(0.3%)	374	(0.1%)
Herpes simplex virus type 1	3386	(0.31%)	2284	(0.31%)	1102	(0.29%)
*Neisseria gonorrhoeae*	6861	(0.62%)	1176	(0.16%)	5685	(1.51%)
*Mycoplasma genitalium*	21,410	(1.94%)	11,686	(1.6%)	9724	(2.59%)
Herpes simplex virus type 2	26,439	(2.39%)	22,816	(3.13%)	3623	(0.96%)
*Chlamydia trachomatis*	31,696	(2.87%)	13,942	(1.91%)	17,754	(4.72%)
*Mycoplasma hominis*	78,593	(7.11%)	63,989	(8.78%)	14,604	(3.88%)
*Candida albicans*	119,899	(10.85%)	118,955	(16.32%)	944	(0.25%)
*Ureaplasma urealyticum*	152,754	(13.82%)	106,191	(14.56%)	46,563	(12.38%)
*Ureaplasma parvum*	312,434	(28.27%)	273,655	(37.53%)	38,779	(10.31%)
*Gardnerella vaginalis*	393,646	(35.62%)	388,004	(53.22%)	5642	(1.5%)

### Positivity rates by age group and sex

**Fig. 1:**
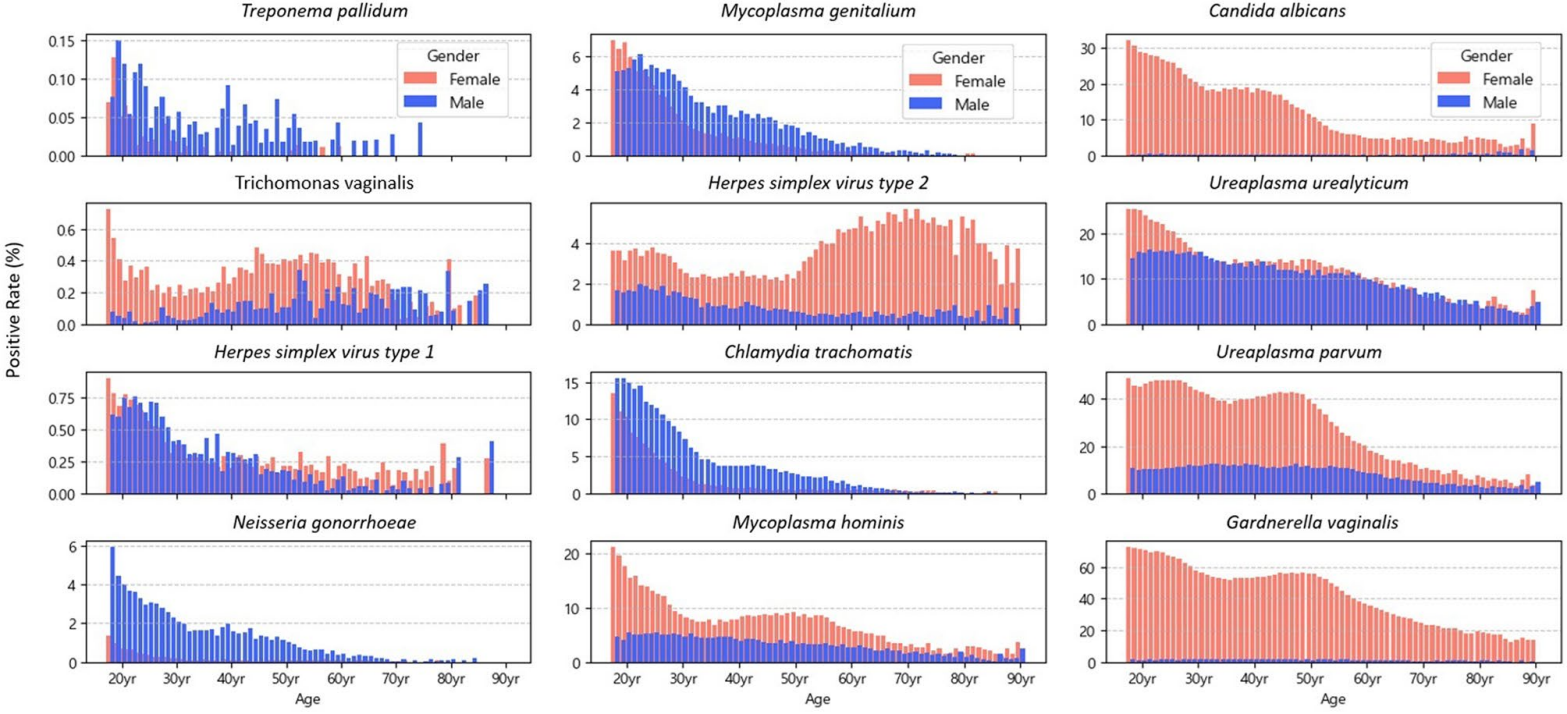
Age-specific positivity rates of STI-related microorganisms by sex, presented using single-year age data

Positivity rates of 12 STI-related microorganisms by sex and age groups are presented in Table 3, which provides the numerical values for direct cross-pathogen comparison. Figure 1 complements these results by illustrating age-related trends using single-year age data.

Among the female samples, most pathogens had the highest positivity rate in the 18–19 age group, with the exception of HSV-2, which presented the highest rates in the older age groups. Additionally, certain pathogens—including *U. urealyticum*,* M. hominis*,* T. vaginalis*,* G. vaginalis*,* and U. parvum*—showed a bimodal pattern, with an initial increase, followed by a decline and a secondary increase around the 50s.

Among the male samples, most organisms presented the highest positivity rates in the 18–29 year age group, followed by a steady decline with increasing age. Notably, *C. trachomatis *(15.55%) and *N. gonorrhoeae *(5.04%) exhibited consistently higher positivity rates in males than in females across all age groups, with peak rates observed at ages 18–19, followed by a steep decline with age. Among individuals under age 30, the positivity rates of *N. gonorrhoeae *(*3.03%*) and *C. trachomatis *(*11.07%*) in males were approximately 7.6-fold and 2.0-fold higher, respectively, than those in females (0.40% and 5.64%, respectively). In contrast, pathogens such as *G. vaginalis* and *C. albicans*, which were more common in females, maintained low positivity rates in males across all age groups, with minimal age-related variation.

**Table 3:** Age-specific positivity rates (%) of 12 sexually transmitted infection (STI)related microorganisms in females and males. Pathogen-related organisms and commensals were grouped based on biological relevance. Within each group, organisms were listed in descending order of total positive cases

Sex	Age group	Pathogen-related organisms							Commensal organisms				
		TP	TV	HSV1	NG	MG	HSV2	CT	MH	CA	UU	UP	GV
Female	18–19	0.11	0.6	0.8	1.1	6.7	3.6	11.9	20.0	31.1	25.3	46.7	72.4
	20–29	0.03	0.3	0.6	0.4	4.1	3.5	5.3	12.8	25.3	20.4	47.1	66.4
	30–39	0.01	0.2	0.3	0.1	1.5	2.4	1.4	7.7	18.8	14.2	40.7	54.1
	40–49	0.00	0.4	0.2	0.1	0.8	2.4	0.6	8.5	16.3	13.8	41.9	54.9
	50–59	0.00	0.4	0.2	0.1	0.3	3.5	0.5	8.2	8.2	12.8	31.1	49.3
	60–69	0.00	0.3	0.2	0.1	0.1	5.0	0.4	5.0	4.8	9.0	15.7	31.8
	70+	0.00	0.1	0.1	0.0	0.0	5.0	0.2	2.6	4.2	5.1	8.5	20.9
Male	18–19	0.12	0.1	0.6	5.0	5.2	1.6	15.6	4.3	0.3	15.4	10.4	1.2
	20–29	0.07	0.0	0.6	2.9	5.2	1.7	10.9	5.1	0.3	15.8	11.2	1.6
	30–39	0.04	0.1	0.3	1.8	3.1	1.1	4.9	4.7	0.3	14.1	12.2	1.7
	40–49	0.04	0.1	0.2	1.4	2.2	0.8	3.5	3.9	0.2	12.4	11.5	1.7
	50–59	0.03	0.2	0.1	0.7	1.1	0.5	2.0	3.2	0.2	11.0	10.6	1.5
	60–69	0.01	0.2	0.1	0.3	0.4	0.5	0.7	2.4	0.1	8.4	6.7	1.0
	70+	0.00	0.2	0.1	0.1	0.1	0.5	0.1	1.4	0.4	5.4	3.7	0.8

Abbreviations: TP, *Treponema pallidum*; TV, *Trichomonas vaginalis*; HSV1, Herpes simplex virus type 1; NG, *Neisseria gonorrhoeae*; MG, *Mycoplasma genitalium*; HSV2, Herpes simplex virus type 2; CT, *Chlamydia trachomatis*; MH, *Mycoplasma hominis*; CA, *Candida albicans*; UU, *Ureaplasma urealyticum*; UP, *Ureaplasma parvum*; GV, *Gardnerella vaginalis*

### Positivity rates by specimen type

To compare the detection rates of the microorganisms by specimen type, we analyzed female vaginal swabs and urine samples. For all pathogens, the odds ratios comparing vaginal swabs to urine samples were greater than 1, indicating higher positivity in vaginal swabs. This difference was statistically significant for all pathogens except *T. pallidum* (Table 4).

**Table 4:** Comparison of positivity rates for 12 sexually transmitted infection (STI)-related microorganisms between vaginal swabs and urine samples from females

Pathogen	Vaginal Positive	Vaginal Negative	Urine Positive	Urine Negative	OR (vaginal vs. urine)	95% CI	*p*-value
*T. pallidum*	66	646,144	3	80,250	2.36	0.81–6.91	0.0829
*T. vaginalis*	2,106	644,104	87	80,166	2.996	2.42–3.71	< 0.001
HSV1	2,163	644,047	86	80,167	3.113	2.51–3.86	< 0.001
*N. gonorrhoeae*	1,089	645,121	77	80,176	1.747	1.39–2.20	< 0.001
*M. genitalium*	11,299	634,911	308	79,945	4.612	4.12–5.17	< 0.001
HSV2	21,419	624,791	1,190	79,063	2.277	2.15–2.41	< 0.001
*C. trachomatis*;	13,290	632,920	556	79,697	3.007	2.76–3.27	< 0.001
*M. hominis*	60,795	585,415	2,845	77,408	2.825	2.72–2.94	< 0.001
*C. albicans*	117,041	529,169	1,509	78,744	11.538	10.96–12.15	< 0.001
*U. urealyticum*	98,780	547,430	6,864	73,389	1.929	1.88–1.98	< 0.001
*U. parvum*	255,671	390,539	16,961	63,292	2.443	2.40–2.49	< 0.001
*G. vaginalis*	363,554	282,656	22,867	57,386	3.228	3.18–3.28	< 0.001

Abbreviations: *T. pallidum*, *Treponema pallidum*; *T. vaginalis*, *Trichomonas vaginalis*; HSV1, Herpes simplex virus type 1; *N. gonorrhoeae*, *Neisseria gonorrhoeae*; *M. genitalium*, *Mycoplasma genitalium*; HSV2, Herpes simplex virus type 2; *C. trachomatis*, *Chlamydia trachomatis*; *M. hominis*, *Mycoplasma hominis*; *C. albicans*, *Candida albicans*; *U. urealyticum*, *Ureaplasma urealyticum*; *U. parvum*, *Ureaplasma parvum*; *G. vaginalis*, *Gardnerella vaginalis*

### Association analysis of coinfections

**Fig. 2:**
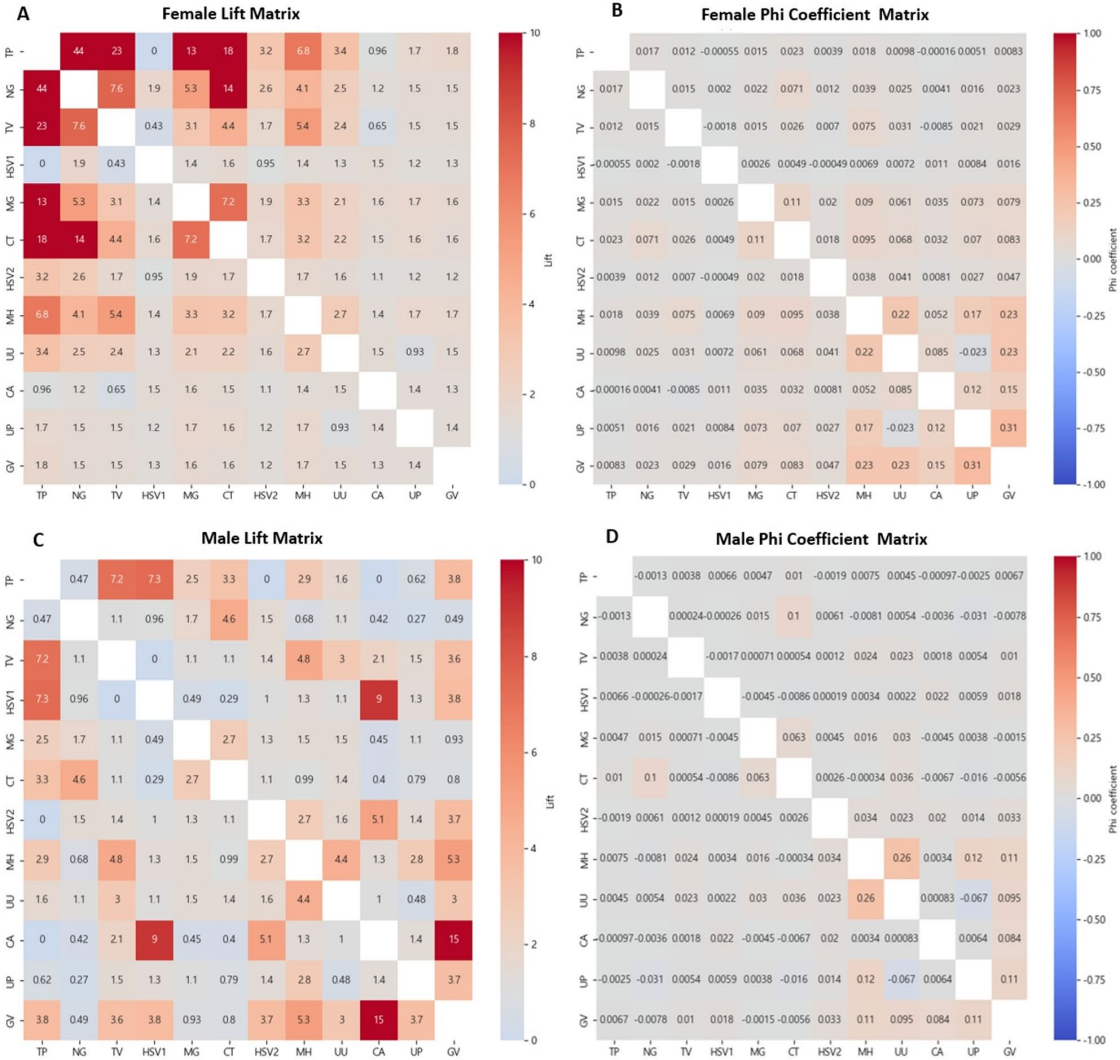
Heatmaps of pairwise pathogen cooccurrences in female (Panels **A**, **B**) and male (Panels **C**, **D**) specimens, based on **lift** (**A**, **C**) and the **phi coefficient** (**B**, **D**). The pathogens in each heatmap were arranged according to the pathogen-specific positivity rates observed within that sex. Abbreviations: TP, *Treponema pallidum*; TV, *Trichomonas vaginalis*; HSV1, Herpes simplex virus type 1; NG, *Neisseria gonorrhoeae*; MG, *Mycoplasma genitalium*; HSV2, Herpes simplex virus type 2; CT, *Chlamydia trachomatis*; MH, *Mycoplasma hominis*; CA, *Candida albicans*; UU, *Ureaplasma urealyticum*; UP, *Ureaplasma parvum;* GV, *Gardnerella vaginalis*

The associations between coinfecting pathogens were assessed using the lift index and phi coefficient and visualized through heatmaps, as shown in Fig. 2. Pathogens were ordered in the heatmaps based on their overall positivity rates, arranged from lowest to highest, to facilitate a visual examination of how positivity rates relate to the strength of association.

Overall, pathogen pairs with lift values greater than 10 were more frequently observed in female samples than in male samples (2 in males and 10 in females). However, the total number of pathogen pairs with lift values ≥3 was comparable between the sexes (32 in males and 34 in females). In both male and female samples, pathogen pairs with a lift index ≥3 included *N. gonorrhoeae–C. trachomatis*, *M. hominis–T. vaginalis*, *T. pallidum–T. vaginalis*, and *T. pallidum–C. trachomatis*, whereas *U. parvum–M. hominis* and *U. urealyticum–M. hominis* presented phi coefficients ≥ 0.1 in both sexes. Lift values tended to be greater in pathogen pairs with low positivity rates, whereas phi coefficients were relatively greater for pathogens with higher overall prevalence rates, such as *G. vaginalis* and *U. parvum. Treponema pallidum* presented high lift values in association with several other pathogens; however, its phi coefficients remained consistently low, mostly below 0.02. For the female samples, the *C. trachomatis–M. genitalium* pair met the criteria of a lift ≥ 3 and a phi coefficient ≥ 0.1. Among the male samples, *M. hominis–G. vaginalis*, *U. parvum–G. vaginalis*, and *U. urealyticum–M. hominis* fulfilled both criteria of a lift ≥ 3 and a phi coefficient ≥0.1. Notably, in the female samples, the combination of *G. vaginalis–U. parvum* had the highest phi coefficient (0.31), whereas its lift value remained relatively low (1.4).

## Discussion

This study assessed epidemiologic patterns of positivity and coinfection by analyzing over one million multiplex PCR results for sexually transmitted infection (STI)-related microorganisms from commercial laboratories. The analysis was performed at the specimen level using data with limited patient-identifying information, and neither clinical symptoms nor repeated tests from the same individual could be considered. As a result, repeated testing in patients with persistent or recurrent symptoms may have increased the observed positivity rates or influenced the coinfection patterns. These limitations reduce the generalizability of the findings and should be taken into consideration when interpreting the results. Nonetheless, a previous study analyzing STI-related microorganisms in Korean women diagnosed with vaginitis during the peri- and postmenopausal periods reported positivity rates of 64.9% for *G. vaginalis*, 38.3% for *U. parvum*, and 20.6% for *C. albicans* [13]. In comparison, the positivity rates in female samples in our study were 53.2% for *G. vaginalis*, 37.9% for *U. parvum*, and 16.3% for *C. albicans*, which are largely consistent with their findings. This consistency suggests that, despite the limitations of our study, the large-scale dataset may still provide reliable insights into pathogen distribution.

In female samples, the positivity rates of several STI-related microorganisms—*G. vaginalis*, *U. parvum*, *U. urealyticum*,* M. hominis*, and *T. vaginalis*—were highest in the teens and those in their twenties, declined with age, and showed a secondary peak around the age of 50. Our analysis, which was based on a large dataset with age stratified by single-year intervals, enabled clearer identification of this bimodal distribution pattern. The bimodal age distribution observed for several pathogens in female samples has also been reported in studies from other countries. PCR testing of vaginal specimens in a commercial laboratory setting in the United States showed that *T. vaginalis* positivity exhibited two distinct peaks at approximately 21–22 years and 48–51 years of age [14]. Additionally, a large-scale study from China involving more than 100,000 women showed a similar bimodal pattern, in which the prevalence of *T. vaginalis* peaked around age 20 and 50 [15]. HPV infection, although not included in our testing panel, has also been reported in multiple countries to display a bimodal age distribution, with an initial peak in young women followed by a second rise in middle to older adulthood [16]. These findings suggest that this age-specific pattern reflects physiological changes in the vaginal environment during the peri- and postmenopausal periods. As estrogen levels decline during menopause, glycogen content in the vaginal epithelium decreases, the epithelium becomes thinner, and vaginal pH increases [17–19]. These changes disrupt the normal *Lactobacillus-*dominant vaginal microbiota, creating an environment that facilitates the overgrowth of various anaerobic bacteria. This explanation is supported by a recent study analyzing vaginal secretions from pre- and postmenopausal women using 16S rRNA gene sequencing, which reported that postmenopausal women more frequently exhibited Community State Types (CSTs) characterized by elevated pH and increased anaerobic bacteria [20]. Such alterations in the vaginal microbial environment may increase susceptibility to infections, including bacterial vaginosis, and could contribute to the resurgence of certain pathogens in women during mid-to-older adulthood.

In our analysis of specimen-specific positivity rates among female patients, vaginal swab samples presented higher detection rates than did urine samples across all pathogens. This difference was statistically significant for all pathogens except *T. pallidum.* This finding is consistent with previous studies reporting superior detection rates in vaginal swabs compared with those in urine samples. Notably, a meta-analysis by Aaron et al. reported higher diagnostic sensitivity of vaginal swabs compared with urine samples for the detection of *C. trachomatis*,* N. gonorrhoeae*, and *T. vaginalis* under paired testing conditions [21].

Multiple coinfections involving different sexually transmitted pathogens have been frequently reported among STI patients. This is primarily attributed to the fact that many STI pathogens share common transmission routes and often cause asymptomatic infections, leading to delayed or no treatment. In the present study, lift, a metric used in association analysis, was applied to quantitatively evaluate coinfection patterns among pathogens. The lift index has been widely used as a measure of association in various epidemiological and clinical studies, including analyses of risk factors for acute myocardial infarction, rheumatoid arthritis, and cancer survival outcomes [22, 23].

In this study, the 12 microorganisms included in the multiplex PCR panel are often tested together in clinical practice; however, they differ substantially in their biological characteristics, transmission patterns, and pathogenicity. *N. gonorrhoeae*,* C. trachomatis*,* M. genitalium*,* T. pallidum*,* T. vaginalis*, and herpes simplex virus are well-established sexually transmitted pathogens, whereas *G. vaginalis*, *U. parvum*, *U. urealyticum*,* M. hominis*, and *C. albicans* are components of the normal genital microbiota or opportunistic microorganisms that may cause infection depending on the host’s physiological or immunological status. Therefore, the associations observed among pathogens in this study should be interpreted with careful consideration of these biological differences. In particular, microorganisms classified as commensals or components of the normal microbiota showed relatively lower lift values compared with classical STI pathogens; however, this should not be interpreted as evidence of weak biological interactions between pathogens. Because commensal organisms generally have a high baseline prevalence, the lift index has an inherent mathematical limitation that restricts large increases, and this characteristic is likely reflected in the observed lower lift values.

In this study, pathogen pairs with lift values ≥3.0 were identified in both male and female samples, specifically, *N. gonorrhoeae–C. trachomatis*, *M. hominis–T. vaginalis*, *T. pallidum–T. vaginalis*, and *T. pallidum–C. trachomatis*. These findings indicate that the probability of codetection of these pathogens is significantly greater than the product of their individual detection probabilities under the assumption of independence, suggesting strong positive associations between them.

Cooccurrence between *N. gonorrhoeae–C. trachomatis* and *M. hominis–T. vaginalis* has been consistently reported in numerous previous studies, suggesting the possibility of epidemiologically meaningful associations. According to earlier studies, *C. trachomatis* infection can induce mucosal damage, activate immune evasion mechanisms, reorganize the host cytoskeleton, and elicit inflammatory responses—ultimately creating a favorable environment for the invasion and proliferation of other pathogens. These interactions have been hypothesized to enhance infectiousness and exacerbate clinical symptoms [24]. Furthermore, in vitro studies have shown that coinfection with *N. gonorrhoeae* and *C. trachomatis* may reduce the number of infectious *Chlamydia* elementary bodies (EBs) compared with *C. trachomatis* monoinfection while maintaining the same level of genome replication. This finding led to the hypothesis that *N. gonorrhoeae* may contribute to the establishment of persistent *C. trachomatis* infection [25]. Our findings support these experimental observations, as *N. gonorrhoeae* and *C. trachomatis* were co-detected at a high frequency in clinical samples.

Additionally, numerous studies have reported frequent coinfections and a potential symbiotic relationship between *M. hominis* and *T. vaginalis*. Hoxha et al. reported that *M. hominis* was detected in 80% of *T. vaginalis*-positive female patients, and *M. hominis* DNA was detected in 10% of cultured *T. vaginalis* isolates, suggesting stable intracellular symbiosis between these two organisms [26]. Similarly, Diaz et al. and van Belkum et al. reported a high prevalence of *M. hominis* in *T. vaginalis*-positive clinical specimens [27, 28]. These findings prompted further investigations into various aspects of the *M. hominis*–*T. vaginalis* interaction, including intracellular survival, transmission potential, pathogenicity, and modulation of host immune responses. Notably, *M. hominis* has been shown to reside and replicate stably within *T. vaginalis* cells, and *T. vaginalis* has been reported to transmit *M. hominis* to uninfected protozoal strains or to human epithelial cells.

Meanwhile, the phi coefficient was employed alongside the lift index to provide a more comprehensive assessment of the associations between binary detection results of pathogens. The phi coefficient is calculated from a 2 × 2 contingency table and is interpreted as a correlation coefficient ranging from − 1 to +1. Unlike lift, which uses only the co-occurrence and individual occurrence frequencies, phi coefficient considers both the presence and absence of pathogens. This makes it particularly useful for evaluating associations between pathogen pairs with substantial differences in prevalence or those with intermediate prevalence levels. Therefore, using the phi coefficient together with lift helps overcome lift’s limitations for low-prevalence pathogens and enables a more reliable assessment of the association by considering all samples. Mathematically, the phi coefficient is equivalent to the Pearson correlation coefficient for binary variables, and both phi and Pearson coefficients have been used in analyses of microbial co-occurrence patterns [29, 30]. However, studies defining an appropriate phi coefficient cut-off for clinically meaningful associations remain limited. In particular, additional comparative studies are needed to clarify the relationship and interpretation between the high clinical plausibility indicated by a lift value greater than 3.0 and the phi coefficient, and this remains an area for future research.

In epidemiological research, commonly used statistical measures for assessing associations, such as relative risk (RR) and odds ratio (OR), are based on the underlying assumption of a causal relationship between exposure and outcome. These metrics are primarily suited for prospective cohort or case‒control study designs. However, their applicability is limited when applied to cross-sectional datasets such as ours, which focus on exploring inter-pathogen relationships based on co-detection rather than causality. In contrast, the lift index and phi coefficient are symmetric statistical measures that allow for bidirectional interpretation of associations between two pathogens. These metrics are particularly useful for investigating co-occurrence or interdependence in situations where clear temporal or causal directionality is absent. The application of diverse analytic methods in the medical field is rapidly increasing with the growing availability of large-scale datasets. Meanwhile, when analyzing clinical data, the temporal or directional relationships between variables are often unclear, and the variables of interest may occur simultaneously. In such non-directional data structures, metrics used in data-mining approaches, such as lift and phi coefficient, serve as useful exploratory tools and can provide foundational information for subsequent in-depth and causal analyses. Accordingly, these metrics are likely to play an increasingly important role in future medical big-data research.

In this study, we constructed heatmaps by arranging pathogens in ascending order of positivity rate to visualize the trends of lift and phi across different prevalence levels. The results showed that lift tended to be higher for pathogen pairs with low prevalence, while phi yielded relatively higher values for pairs with higher prevalence. Notably, *T. pallidum* demonstrated consistently high lift values across multiple pathogen combinations, yet its phi values remained uniformly low. This suggests that lift is more sensitive to detecting co-occurrence among low-prevalence pathogens, whereas phi is more strongly influenced by the overall distribution, including negative cases [29, 31, 32].

An important consideration when interpreting associations among sexually transmitted pathogens is whether the diagnostic method accurately reflects the underlying infection status. For *T. pallidum*, serologic testing remains the primary reference standard for syphilis diagnosis, whereas the diagnostic performance of PCR varies considerably depending on specimen type and disease stage, with reported sensitivities ranging from approximately 15% to 50% across studies [33, 34]. PCR assays performed on urine or vaginal swab specimens primarily reflect the presence of organisms at the sampled mucosal site rather than systemic infection status. Because *T. pallidum* infection frequently involves systemic dissemination beyond the primary lesion, negative PCR results obtained from non-lesion specimens do not exclude infection. Similarly, for herpes simplex virus, viral detection depends on the level of viral shedding at the sampled site, and a negative PCR result does not necessarily exclude infection. These limitations indicate that analyses based solely on PCR results may not fully reflect true infection, underscoring the need for further integrative studies that incorporate multiple diagnostic modalities.

The high frequency of coinfection and the age-specific variations in pathogen positivity rates observed in this study provide epidemiological insights that may contribute to a better understanding of national STI testing and management frameworks. These findings may serve as a basis for future research and policy discussions regarding age-targeted prevention strategies, screening program design, and the composition of STI testing panels within the National Health Insurance system. However, because this study was an exploratory analysis based on cross-sectional specimen-level data with limited clinical information, the findings should be interpreted cautiously and regarded as hypothesis-generating.

At present, robust comparative studies remain limited regarding which co-occurrence metrics best reflect true biological or clinical relationships. Therefore, careful interpretation is essential when these metrics are used, with attention paid to their mathematical properties, limitations, and validation across diverse populations and study settings. Compared with single infections, coinfections of sexually transmitted pathogens have been reported to result in more adverse clinical outcomes, including increased transmissibility, more severe symptoms, a greater risk of complications, and reduced effectiveness of treatment and prevention. These findings suggest that coinfections involve complex biological interactions that influence pathogenicity and treatment responses. Therefore, a deeper understanding and a more systematic approach to coinfection analysis are essential for the effective management and treatment of STIs, and the present study provides foundational evidence to support future research and policy discussions in this area.

## Data Availability

The dataset analyzed in this study comprises over one million de-identified clinical specimens collected through routine diagnostics at a commercial laboratory. Due to patient confidentiality and institutional policies, the full dataset is not publicly available. However, summary data supporting the findings of this study are available from the corresponding author upon reasonable request.
